# Successful mechanical thrombectomy and stent exclusion of sacral chordoma tumor thrombus

**DOI:** 10.1016/j.radcr.2022.09.082

**Published:** 2022-11-18

**Authors:** Valeria Gioioso, David Duncan, Jeet Minocha, Jonas Redmond

**Affiliations:** University of California San Diego Health, 200 W Arbor Dr, San Diego, CA 92103 USA

**Keywords:** Interventional radiology, Sacral chordoma, Mechanical thrombectomy, Tumor thrombus, Deep venous thrombosis, Venous stenting

## Abstract

A 77-year-old man with history of sacral chordoma and pulmonary embolism presented to the emergency room with a 1-day history of diffuse left flank and lower extremity swelling. The patient was found to have thrombus in the left common and external iliac veins. The patient was brought to Interventional Radiology for mechanical thrombectomy using the Inari ClotTriever and a sample of extracted thrombus was sent to pathology. Analysis on the sample was positive for sacral chordoma, consistent with tumor thrombus. The patient returned after 6 weeks with similar symptoms and repeat mechanical thrombectomy was performed with the Inari ClotTriever and stent placement through the left common and external iliac vein with an Ovation iX stent graft. The patient remained asymptomatic following the second procedure at repeat follow-up at 6 weeks.

## Introduction

Arising from notochord remnants, chordomas are rare, slow-growing malignancies that have varied clinical presentations due to gradual invasion and destruction of adjacent structures [1]. Vascular involvement is associated with poorer overall survival of sacral chordoma [2]. As in other malignancies with vascular involvement, presence of bland, or tumor thrombus may guide treatment options. Imaging studies (eg, contrast-enhanced MRI) may help differentiate bland from tumor thrombus [3]. This case report describes the use of the Inari ClotTriever (Inari Medical, Irvine, CA) device for extraction of initially unknown tumor thrombus, the repeat use of the device for known tumor thrombus, and the subsequent placement of a stent-graft to prevent future intravascular tumor ingrowth. This report was exempted from institutional review board approval.

## Case report

A 77-year-old man presented to the emergency room with a 1-day history of diffuse left flank and lower extremity swelling. The patient denied symptoms of neurovascular compromise but endorsed left lower extremity tightness and difficulty with movement. He had a history of sacral chordoma which was diagnosed 5 years prior to presentation, for which he underwent 2 partial surgical resections within 6 months following diagnosis and 4 courses of proton beam radiation therapy, the last of which was completed 7 months prior to presentation. The patient's history was also significant for pulmonary embolism diagnosed 5 months prior to presentation, for which he was on apixaban.

**Fig. 1:**
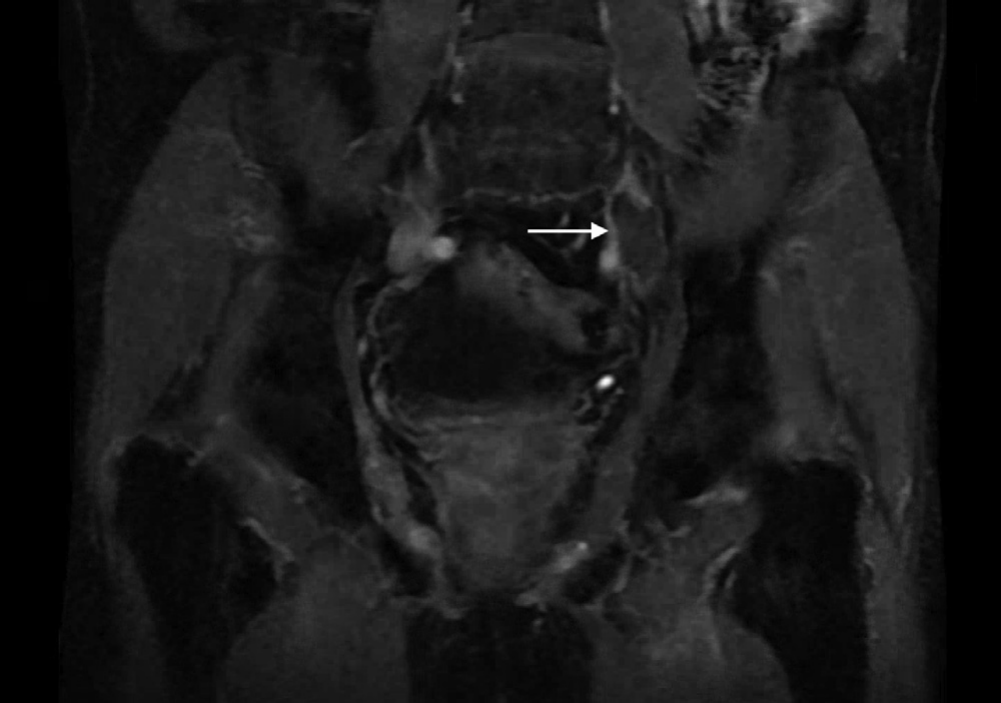
Coronal LAVA postcontrast demonstrating thrombus in left common iliac vein with lack of internal enhancement.

Ultrasound of the lower extremities was negative for acute deep venous thrombosis (DVT) in the bilateral lower extremities. CT of the abdomen and pelvis demonstrated near-occlusive thrombus in the left common iliac vein (CIV) with extension of thrombus into the left external and internal iliac veins (EIV, IIV). An MRI could not differentiate between bland and tumor thrombus due to lack of definite enhancement, although tissue invasion from the chordoma was noted (Fig. 1).

Interventional radiology (IR) was consulted for evaluation and management. Following a complete discussion regarding the risks, benefits, and alternatives of intervention, the patient consented to percutaneous left iliac vein thrombectomy for symptomatic relief and inferior vena cava (IVC) filter placement for presumed failure of anticoagulation.

**Fig. 2:**
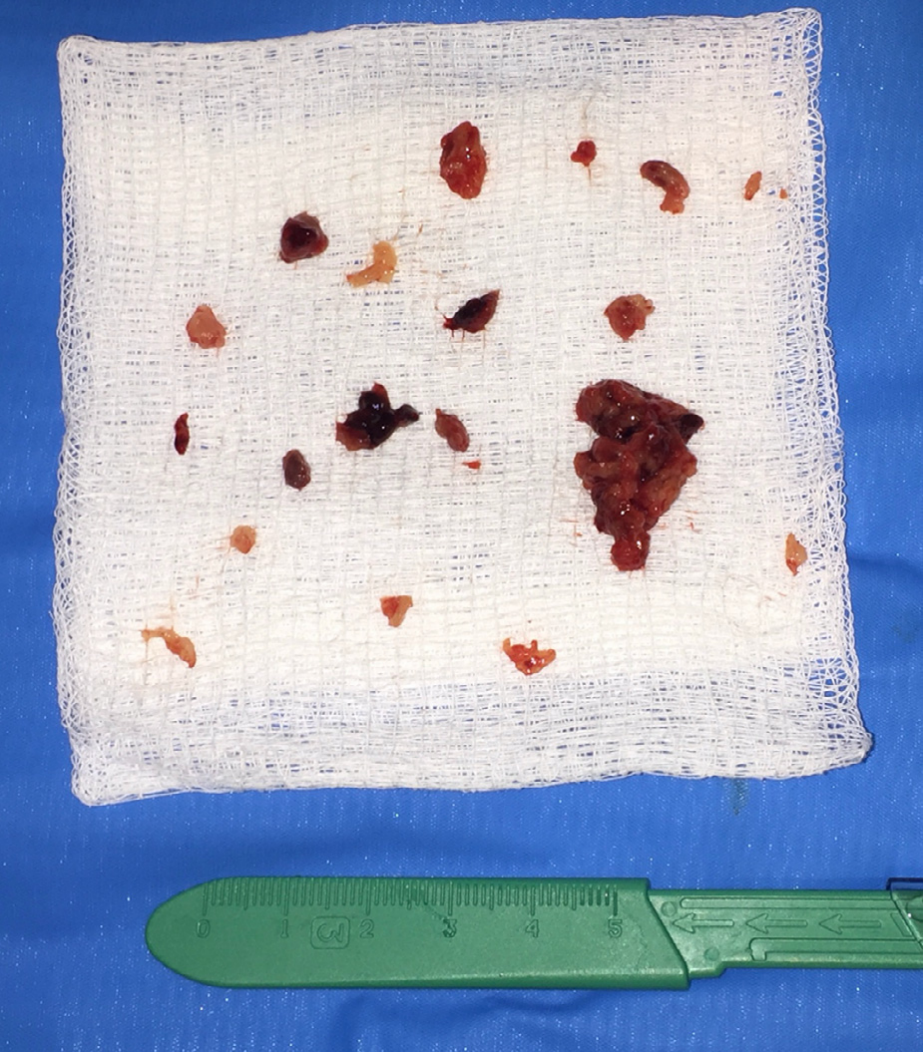
Extirpated matter from the first mechanical thrombectomy procedure using the Inari ClotTriever catheter.

**Fig. 3:**
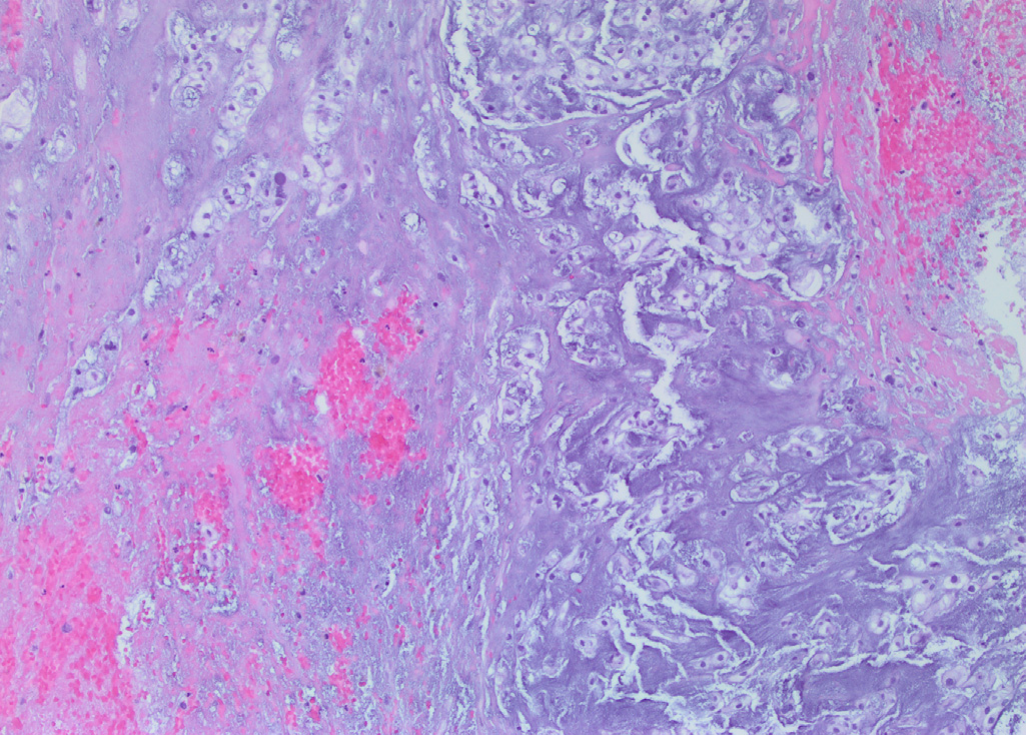
Hematoxylin and eosin staining of submitted specimen under 100× microscopy demonstrating myxoid matrix with nests of tumor cells with a physaliphorous, or bubbly and vacuolated, appearance. In the background there is fibrin and necrosis. Sample conclusive for sacral chordoma.

For the procedure, the left greater saphenous vein was accessed and diagnostic venography confirmed near-complete occlusion of the left EIV and CIV with absence of reflux into the left IIV, compatible with prior imaging. After upsizing the percutaneous vascular access sheath, the Inari ClotTriever device was positioned through the area of occlusion and into the IVC. Two passes were made, passing the coring element of the device from the IVC through the area of occlusion and into the left EIV. Mixed bloody, tan, and white fibrinous material was captured in the collection bag of the ClotTriever catheter. Follow-up venography demonstrated resolution of the previous filling defect in the left CIV with no underlying stenosis. The extracted specimens were placed into formalin and submitted to pathology (Fig. 2). Following thrombectomy, a Denali IVC (Bard Peripheral Vascular, Inc, Tempe, AZ) filter was deployed in the infrarenal IVC. The patient tolerated the procedure well and reported significant improvement in symptoms by postprocedure day 5. The final pathologic diagnosis of the specimen obtained from the left CIV was conclusive for sacral chordoma (Fig. 3).

**Fig. 4:**
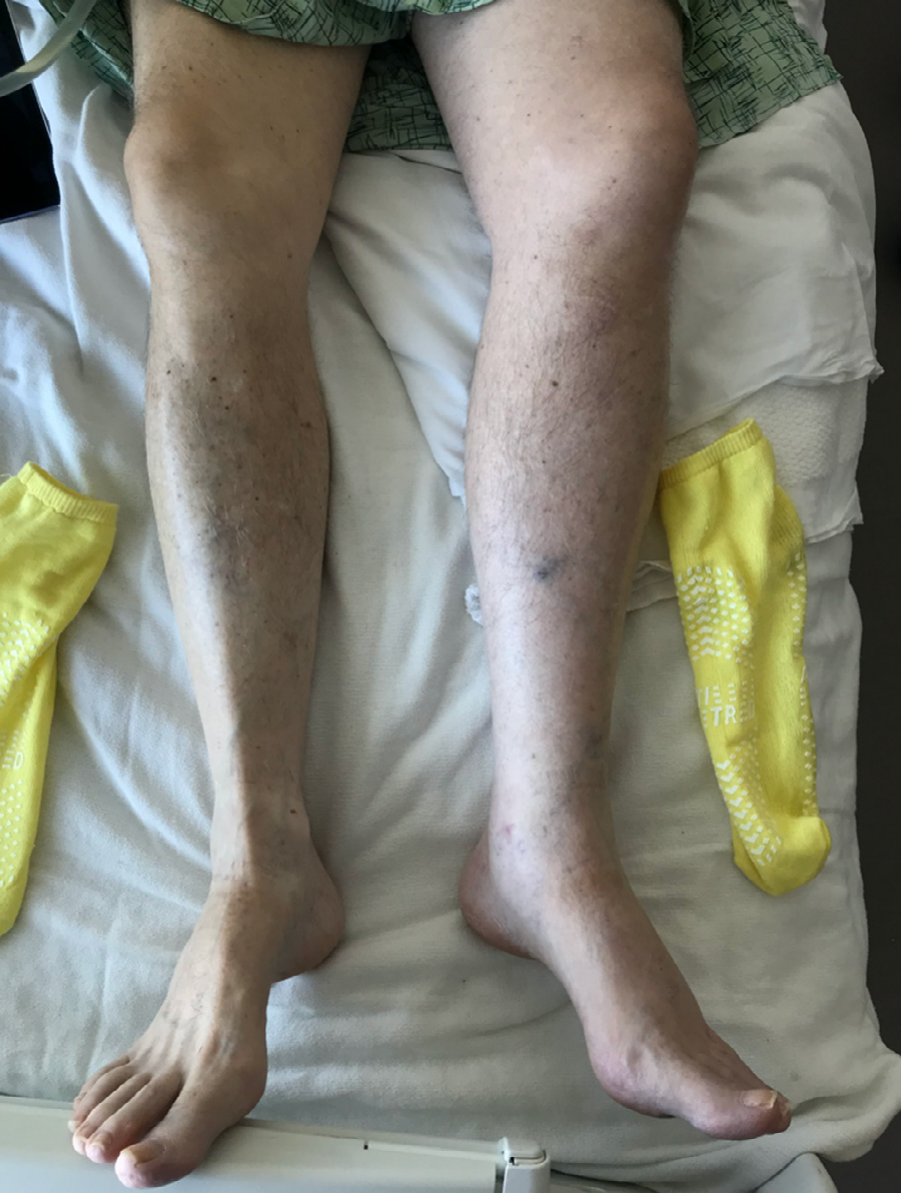
Picture of patient's legs with recurrent symptoms of swelling and pain, prior to second thrombectomy with stent placement.

**Fig. 5:**
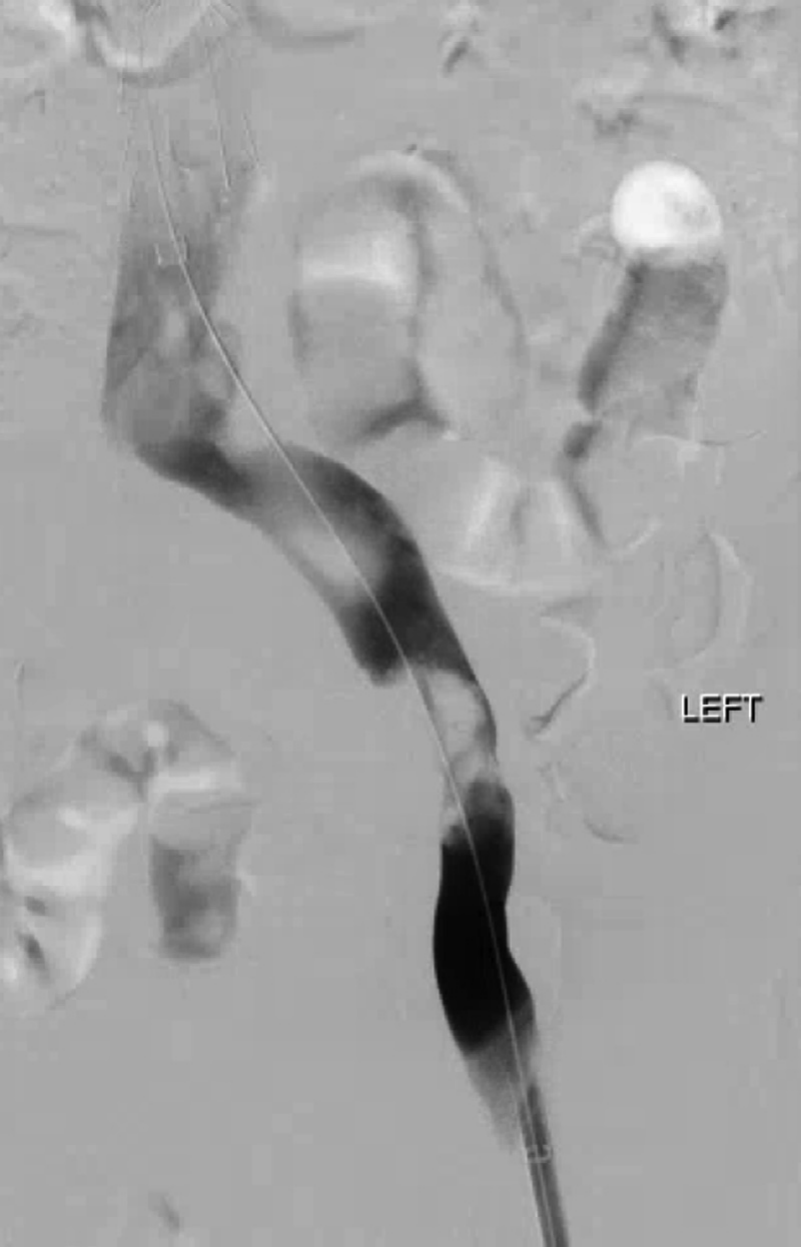
Image during second mechanical thrombectomy and stent placement. Initial venogram demonstrating recurrent occlusion in left CIV/EIV.

**Fig. 6:**
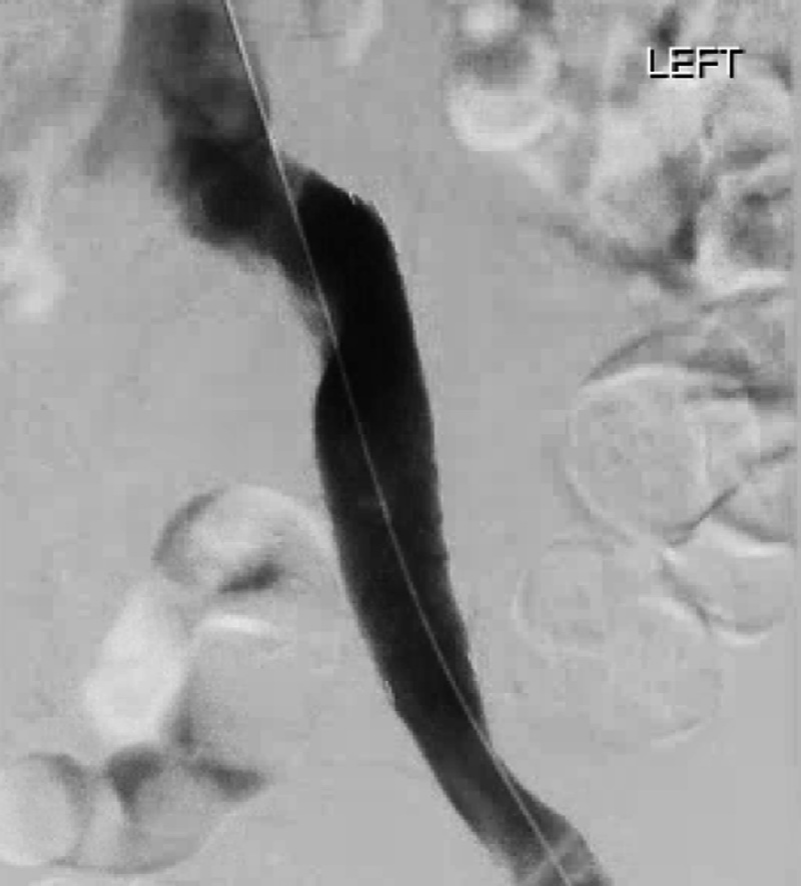
Image during second procedure. Improved flow following thrombectomy and stent placement without residual stenosis or flow limitation.

Six weeks later, the patient experienced recurrent left lower extremity swelling (Fig. 4). Lower extremity Doppler was again negative for DVT, but a CT abdomen and pelvis demonstrated recurrent occlusion of the left CIV and EIV. Suspecting recurrent tumor thrombus on account of the pathology result from the first procedure, the patient agreed to and underwent repeat percutaneous thrombectomy with stent-graft placement to decrease the risk of recurrent vascular ingrowth of tumor and improve the lifestyle limitations resulting from the lower extremity swelling. The second procedure was performed in a similar manner. Venography confirmed thrombus in the left CIV and EIV (Fig. 5). To prevent entanglement with the indwelling IVC filter, right internal jugular access was also obtained, allowing deployment of the ClotTriever device within a 16 Fr jugular sheath positioned with the tip immediately below the IVC filter. Thrombectomy was performed with a total of 5 passes of the ClotTriever device. Via the right internal jugular vein access, a tapered 18 mm to 14 mm diameter × 80 mm length Ovation iX stent graft (Endologix, Irvine, CA) was then deployed within the left CIV and EIV and angioplastied with 16 and 18 mm balloons. Completion venography demonstrated a patent stent-graft without evidence of residual filling defects or stenosis (Fig. 6). The patient was discharged on postprocedure day 3. Pathology on the submitted specimen again confirmed sacral chordoma. At follow-up 6 weeks after the second procedure, the patient remained symptom-free.

## Discussion

Prior to the first procedure and pathologic confirmation on the extracted material, the differential etiology for this patient's thrombus included malignancy-associated hypercoagulable state, postradiation vascular occlusion, or tumor-in-vein. Each is treated according to their underlying pathophysiology. Bland thrombus may be treated with anticoagulation or thrombolysis whereas post-radiation vascular occlusion may be treated with stenting. However, successful treatment of tumor thrombus is often unrealized, whether from non-response to anticoagulation, prior lack of sufficient tools for thrombectomy, or the concern for tumor embolization during manipulation and stenting.

In the present case, the patient's recurrent thrombus development despite anticoagulation suggests an underlying tumor etiology, though the MRI was inconclusive. For this reason, during the first mechanical thrombectomy procedure, the removed material was sent to pathology and was diagnostic for malignancy. In a similar, previously described case, the Inari ClotTriever device was used for extraction of IVC thrombus. The extracted material in that case was confirmed by pathology to be liposarcoma, compatible with the patient's history [4]. These cases demonstrate the utility of specialized mechanical thrombectomy devices such as the Inari ClotTriever for both diagnostic and therapeutic purposes.

The Inari ClotTriever device, described for its safety, efficacy, and ease of use in DVT, may serve a role in restoring flow and improving patients’ symptoms from malignant venous occlusion. Suspicion of tumor thrombus should not deter an interventional radiologist from attempting to achieve clinically significant quality of life improvements. The addition of covered stent placement following thrombectomy can additionally extend patients’ relief from symptoms by preventing recurrence due to tumor ingrowth.

## Patient consent

This report was exempted from institutional review board approval. Informed consent was obtained. All submitted images have been de-identified.
